# Tirzepatide-Associated Severe Hepatocellular Injury: A Case Report and Diagnostic Considerations

**DOI:** 10.3390/diagnostics16152359

**Published:** 2026-07-27

**Authors:** Dae Gon Kim, Jeong-Ju Yoo, Sang Gyune Kim, Young Seok Kim

**Affiliations:** Division of Gastroenterology and Hepatology, Department of Internal Medicine, Soonchunhyang University Bucheon Hospital, Bucheon 14584, Republic of Korea; gon0704@hanmail.net (D.G.K.); mcnulty@schmc.ac.kr (S.G.K.); liverkys@schmc.ac.kr (Y.S.K.)

**Keywords:** tirzepatide, drug-induced liver injury (DILI), hepatotoxicity, rapid weight loss, GLP-1 receptor agonist, case report

## Abstract

**Background/Objectives:** Tirzepatide, a dual glucose-dependent insulinotropic polypeptide and glucagon-like peptide-1 receptor agonist, is increasingly used for glycemic control and weight reduction and has a generally favorable hepatic safety profile. However, rare cases of clinically significant drug-induced liver injury have been reported. We report a case of severe hepatocellular injury during tirzepatide therapy and compare it with previously reported cases. **Case Presentation:** A 36-year-old man developed gastrointestinal symptoms, dark urine, and severe hepatocellular injury approximately 5 weeks after starting tirzepatide for weight reduction, shortly after dose escalation from 2.5 mg to 5 mg weekly and amid rapid weight loss of approximately 10 kg. Extensive evaluation excluded viral, autoimmune, metabolic, biliary, and structural causes, with no competing medication or supplement exposure identified. Tirzepatide was discontinued, and clinical and biochemical status improved rapidly. The Roussel Uclaf Causality Assessment Method (RUCAM) score was 7, indicating probable drug-induced liver injury. **Discussion:** Previously reported cases demonstrate heterogeneous biochemical patterns and severity, ranging from mild hepatocellular injury to mixed or cholestatic presentations and acute liver failure requiring transplantation. The mechanism remains uncertain. An idiosyncratic drug reaction is the most plausible explanation, while rapid weight loss, dose escalation, and hepatic steatosis may represent susceptibility modifiers rather than established causes. **Conclusions:** Tirzepatide-associated liver injury appears rare but can be clinically severe. Clinicians should remain alert to the possibility of drug-induced liver injury when unexplained hepatic symptoms or liver biochemical abnormalities arise during treatment.

## 1. Introduction

Tirzepatide is a dual glucose-dependent insulinotropic polypeptide (GIP) and glucagon-like peptide-1 (GLP-1) receptor agonist that has demonstrated substantial efficacy in glycemic control and weight reduction in patients with type 2 diabetes and obesity [1,2]. Given the growing global burden of metabolic dysfunction-associated steatotic liver disease (MASLD), pharmacologic agents that promote significant weight loss have attracted considerable attention for their potential to improve hepatic steatosis and metabolic profiles [3].

Clinical trials have shown that tirzepatide not only reduces body weight but also improves liver-related parameters, including reductions in hepatic fat content and serum aminotransferase levels [3,4,5,6,7]. Accordingly, it has been considered a promising therapeutic option for metabolic liver disease patients. While gastrointestinal adverse effects are common, clinically significant hepatotoxicity has rarely been reported [8]. Nevertheless, an increasing number of cases of tirzepatide-associated drug-induced liver injury (DILI) have been reported, with heterogeneous latency, biochemical patterns, and severity ranging from mild hepatocellular injury to mixed or cholestatic presentations and acute liver failure [9,10,11,12,13,14,15,16,17,18,19,20].

Diagnosis remains challenging, as patients receiving tirzepatide may have obesity, metabolic risk factors, underlying hepatic steatosis, rapid treatment-related weight loss, or concomitant medications that complicate interpretation of liver biochemical abnormalities [9,10,11,12,13,14,15,16,17,18,19,20]. Careful assessment of exposure timeline, biochemical pattern, alternative etiologies, dechallenge response, and causality is therefore essential in suspected cases [10,21,22,23].

Here, we describe the clinical course and causality assessment of early-onset severe hepatocellular injury occurring during tirzepatide therapy, shortly after dose escalation and amid rapid weight loss. The clinical contribution of this case lies in its clearly documented pretreatment liver biochemistry, exposure timeline, structured causality assessment, and prompt improvement after drug withdrawal. We also compare it with previously reported cases to place it within the broader clinical spectrum of tirzepatide-associated DILI, focusing on clinical features, causality assessment, management, and outcomes. This case report was prepared in accordance with the CARE guidelines, and the completed CARE checklist is provided as Supplementary Materials.

## 2. Case Presentation

A 36-year-old man presented to the emergency department with a 3-day history of nausea and dyspepsia, followed by vomiting beginning one day prior to admission. He also reported dark urine.

He had no significant past medical history except for a tonsillectomy performed 15 years earlier. There was no recent travel history or family history of liver disease. He was taking no prescription or over-the-counter medications, including acetaminophen or nonsteroidal anti-inflammatory drugs, and denied use of herbal products, dietary supplements, or recreational drugs. His alcohol consumption was minimal, averaging approximately one bottle of soju per month.

For weight reduction, he had initiated weekly tirzepatide injections on 2 March 2026, receiving four doses of 2.5 mg before dose escalation to 5 mg on 30 March 2026. His baseline body weight was 92 kg, and he lost 10 kg over approximately 5 weeks.

At presentation, his height was 175 cm and weight was 82 kg (body mass index, 26.8 kg/m^2^). Vital signs were stable, with blood pressure of 131/70 mmHg, heart rate 85 beats per minute, respiratory rate 20 breaths per minute, and body temperature 36.8 °C. Physical examination revealed scleral icterus. The abdomen was soft and flat, with mild epigastric tenderness but no rebound tenderness. Bowel sounds were normal.

Laboratory tests performed two days before tirzepatide initiation showed a normal alanine aminotransferase (ALT) level of 19 U/L. However, at presentation, laboratory evaluation revealed marked elevation of liver enzymes, with aspartate aminotransferase (AST) 1336 U/L, ALT 2626 U/L, and total bilirubin 4.0 mg/dL (indirect bilirubin 2.5 mg/dL). Additional findings included gamma-glutamyl transferase 118 U/L, albumin 4.8 g/dL, alkaline phosphatase (ALP) 91 U/L, prothrombin time 20.1 s (international normalized ratio, INR 1.75), and activated partial thromboplastin time 39.8 s, consistent with a predominant pattern of hepatocellular injury.

**Figure 1:**
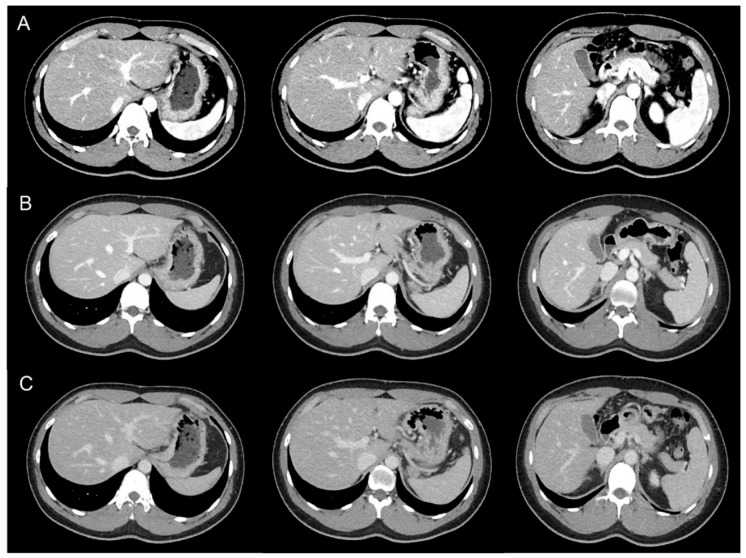
Contrast-enhanced dynamic liver CT showing diffuse, moderate hepatic steatosis without focal lesions. (**A**) Arterial phase, (**B**) portal venous phase, and (**C**) delayed phase.

Serologic testing for viral hepatitis was negative, including hepatitis A virus immunoglobulin M (IgM), hepatitis B surface antigen, hepatitis B core IgM antibody, hepatitis C antibody, and hepatitis E virus IgM antibody. Testing for other viral infections, including human immunodeficiency virus, Epstein–Barr virus, herpes simplex virus, and cytomegalovirus, was also negative. Autoimmune markers, including antinuclear antibody, anti-smooth muscle antibody, anti-liver–kidney microsomal antibody, and antimitochondrial antibody, were also negative. Serum immunoglobulin G and ceruloplasmin levels were normal. Contrast-enhanced dynamic liver computed tomography demonstrated diffuse moderate hepatic steatosis without focal hepatic lesions, with no abnormalities in gallbladder, biliary tree, pancreas, or spleen (Figure 1). Abdominal ultrasonography and magnetic resonance cholangiopancreatography were not performed because the predominantly hepatocellular biochemical pattern, normal ALP level, and absence of biliary abnormalities on computed tomography (CT) suggested a low likelihood of clinically significant biliary obstruction.

With no alternative etiologies, the Roussel Uclaf Causality Assessment Method (RUCAM) score was 7, indicating probable tirzepatide-associated DILI. Tirzepatide was discontinued, and the patient was managed with supportive care and close monitoring of liver function tests. Liver enzymes began decreasing by hospital day 2 (AST 633 U/L, ALT 1740 U/L, total bilirubin 2.96 mg/dL), with further improvement by day 6 (AST 153 U/L, ALT 596 U/L, total bilirubin 1.07 mg/dL). Clinical symptoms also improved, and he was discharged with outpatient follow-up.

**Figure 2:**
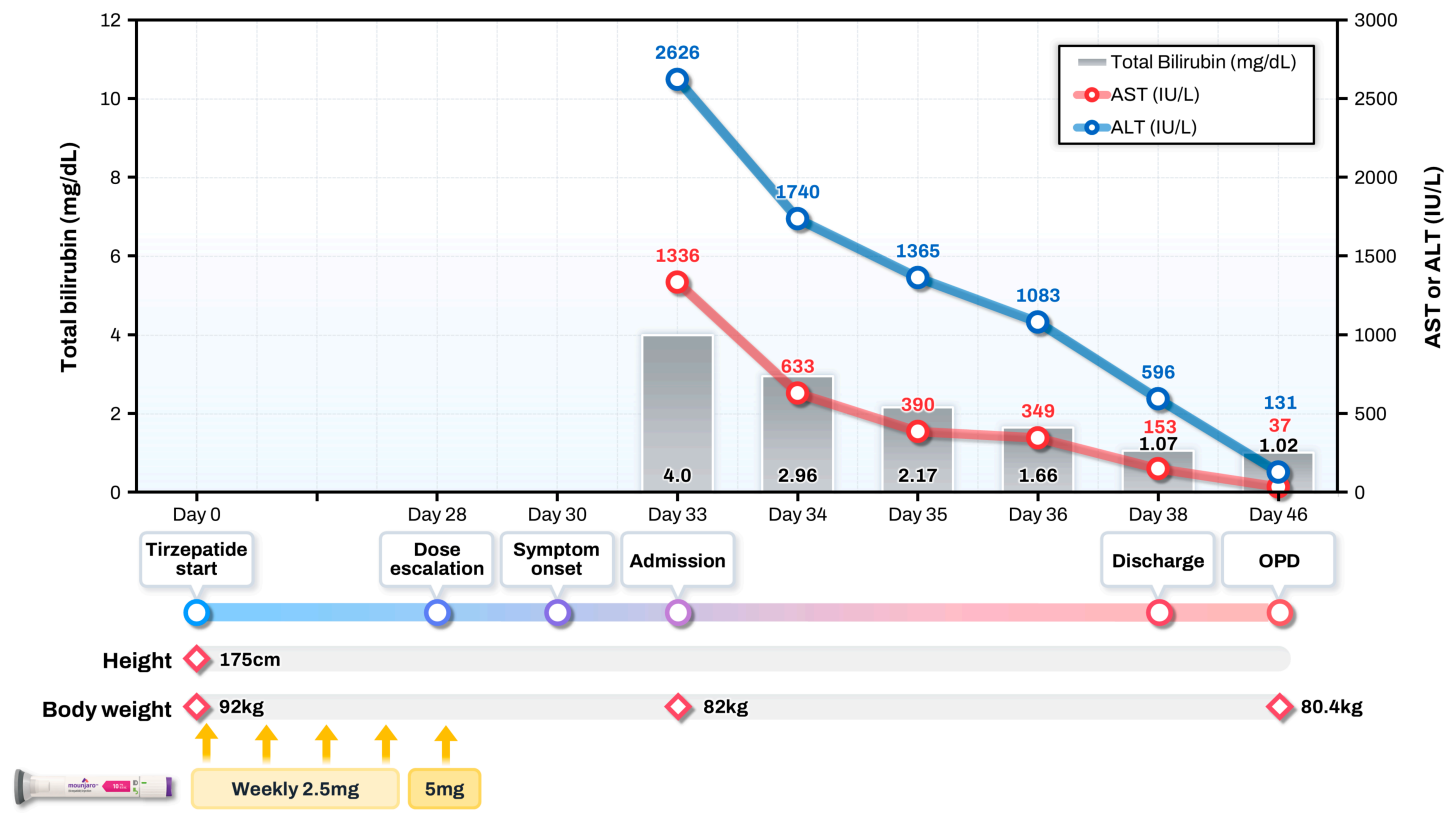
Clinical course of acute hepatocellular injury following tirzepatide therapy. Serial changes in serum liver enzymes and total bilirubin are shown in relation to key clinical events. After tirzepatide initiation and subsequent dose escalation, the patient developed symptoms followed by marked elevation of aminotransferases (peak AST 1336 U/L, ALT 2626 U/L) and total bilirubin (peak 4.0 mg/dL), leading to hospital admission. Following tirzepatide discontinuation and supportive care, liver function tests gradually normalized. The timeline also demonstrates progressive weight reduction from 92 kg to 80.4 kg during treatment.

At 14 days after admission, follow-up laboratory tests showed continued improvement, with AST 37 U/L, ALT 131 U/L, and total bilirubin 1.02 mg/dL (indirect bilirubin 0.8 mg/dL). His body weight had decreased further to 80.4 kg, and he remained clinically stable without additional treatment. A timeline diagram illustrates the temporal relationship between tirzepatide administration, symptom onset, hospital presentation, body weight changes, and laboratory trends (Figure 2).

### Patient Perspective

The patient was initially satisfied with the rapid weight loss achieved during tirzepatide therapy and experienced no significant discomfort during the first several weeks. After dose escalation, he developed upper abdominal discomfort, nausea, dark urine, and vomiting, and grew concerned upon learning that he had severe liver injury. He was reassured as his symptoms and liver biochemical abnormalities improved after tirzepatide discontinuation. At follow-up, he remained asymptomatic and stated that he would not use tirzepatide again.

## 3. Discussion

### 3.1. Diagnostic Assessment and Causality

In this report, we describe a case of severe hepatocellular injury occurring approximately 5 weeks after tirzepatide initiation, shortly after dose escalation and amid rapid weight loss. The temporal relationship between drug exposure and symptom onset, exclusion of competing etiologies, and prompt biochemical improvement after tirzepatide discontinuation supported a diagnosis of tirzepatide-associated DILI. The RUCAM score was 7, indicating probable causality (Table 1) [24]. Liver biopsy was deferred given the prompt clinical and biochemical improvement after tirzepatide withdrawal, extensive exclusion of competing etiologies, absence of persistent liver injury or signs of autoimmune-like hepatitis, and the low likelihood that histologic findings would alter management. Nevertheless, the absence of histologic confirmation limits definitive characterization of the injury pattern and assessment of the potential contribution of the moderate hepatic steatosis observed on CT.

### 3.2. Clinical Spectrum of Reported Tirzepatide-Associated Liver Injury

Tirzepatide has demonstrated substantial efficacy in glycemic control and weight reduction, along with favorable hepatic effects, including reductions in hepatic fat content and serum aminotransferase levels [1,2,3,4,5,6,7]. Similarly, GLP-1 receptor agonists such as semaglutide and liraglutide have demonstrated favorable hepatic effects in patients with steatohepatitis and metabolic liver disease [5,6,7,25,26]. These findings indicate that GLP-1–based therapies are generally associated with hepatic improvement rather than predictable hepatotoxicity. Nevertheless, an increasing number of clinically significant tirzepatide-associated liver injury cases have been reported, suggesting rare adverse reactions may occur in susceptible individuals [9,10,11,12,13,14,15,16,17,18,19,20]. This apparent paradox raises the possibility that individual susceptibility or rapid metabolic change may modify liver injury risk in selected patients. However, the rarity and unpredictability of reported cases are more consistent with an idiosyncratic reaction than a class-mediated or dose-dependent toxic effect [27].

As summarized in Table 2, previously reported cases have shown heterogeneous latency, biochemical patterns, severity, and outcomes [9,10,11,12,13,14,15,16,17,18,19,20]. Hepatocellular injury has been the predominant presentation, though mixed injury and one case of cholestatic hepatitis have also been described. Clinical severity has ranged from mild aminotransferase elevation to acute liver failure requiring transplantation. Within this heterogeneous clinical spectrum, the present case provides detailed documentation of early-onset severe hepatocellular injury with jaundice and coagulopathy following a clearly defined tirzepatide exposure and dose-escalation timeline. Its clinical contribution lies in the availability of a normal pretreatment ALT level, rapid weight loss of approximately 10 kg over 5 weeks, extensive exclusion of competing causes, structured causality assessment, and prompt improvement after drug withdrawal.

### 3.3. Potential Mechanisms and Susceptibility Modifiers

Rapid weight loss may have contributed to individual susceptibility in the present case, though a direct causal relationship cannot be established. The patient lost approximately 10 kg over 5 weeks, and liver injury developed shortly after the weekly tirzepatide dose was increased from 2.5 mg to 5 mg. Rapid weight reduction and severe caloric restriction can increase adipose tissue lipolysis and hepatic delivery of free fatty acids, potentially imposing metabolic stress on the liver [28,29,30]. Excessive fatty acid flux has also been associated with oxidative stress and mitochondrial dysfunction in experimental and clinical settings [28,31]. These mechanisms, however, have not been directly demonstrated in tirzepatide-associated DILI and are largely extrapolated from studies of profound weight loss, malnutrition, and aggressive steatohepatitis. Several previously reported cases also documented substantial weight loss before liver injury, suggesting rapid metabolic change may have modified susceptibility in some patients [11,19]. Therefore, rapid weight loss, dose escalation, and underlying hepatic steatosis should be regarded as possible susceptibility modifiers rather than established causes of liver injury.

### 3.4. Clinical Implications and Monitoring

Tirzepatide-associated liver injury should be considered when patients develop persistent or worsening gastrointestinal symptoms accompanied by dark urine, jaundice, or unexplained liver biochemical abnormalities during treatment. Current evidence is insufficient to support routine serial monitoring in all patients; however, baseline liver biochemical tests, including ALT, AST, ALP, and total bilirubin, may provide a useful reference for subsequent evaluation. Repeat testing should be considered when clinical signs suggest hepatic injury, and prothrombin time or the INR should be assessed when clinically significant liver injury is suspected. Particular attention may be warranted after dose escalation or substantial treatment-related weight loss, though these factors have not been established as predictors of DILI. Prompt evaluation for alternative causes, discontinuation of the suspected agent, and supportive care remain the mainstays of management [21,22,23]. Further studies are needed to clarify the underlying mechanisms and identify factors associated with individual susceptibility.

## 4. Conclusions

We report a case of severe hepatocellular injury occurring during tirzepatide therapy, shortly after dose escalation and amid rapid early weight loss. Although tirzepatide generally has a favorable hepatic safety profile, rare cases of clinically significant liver injury are increasingly being recognized. The underlying mechanism remains uncertain, and rapid weight loss, dose escalation, and hepatic steatosis should be regarded as possible susceptibility modifiers rather than established causes or triggers. Clinicians should consider tirzepatide as a possible cause when unexplained hepatic symptoms or liver biochemical abnormalities arise during treatment, and should promptly evaluate alternative causes and discontinue tirzepatide when clinically significant liver injury is suspected. This report is limited by its single-case design, the absence of liver biopsy, and the lack of rechallenge. Further accumulation of well-characterized cases is needed to clarify susceptibility factors and inform future monitoring strategies.

## Figures and Tables

**Table 1:** Roussel Uclaf Causality Assessment Method (RUCAM) Score.

Factor	Case	Score
Time of onset from beginning of drug use	5–90 days	2
Change in ALT between peak and stopping of the drug use	Decrease ≥ 50% within 8 days	3
Risk factors		
1. Age > 55	No	0
2. Alcohol	No	0
Concomitant drug use	No concomitant drugs	0
Exclusion of other causes	All group I causes ruled out	1
Previous hepatotoxicity information	Reaction published but unlabeled	1
Response to readministration	Not readministered	0
Total score		7 (Probable)

**Table 2:** Reported Cases of Tirzepatide-Associated Drug-Induced Liver Injury: A Literature Review.

Case	Clinical Background and Concomitant Medications	Dose and Duration of Tirzepatide	Weight Change	Pattern and Peak Laboratory Values	RUCAM	Management	Time to Resolution	Outcome
Sohal 2024 (37/F) [9]	Obesity, hypertension, and hypercholesterolemia; ethinyl estradiol/levonorgestrel	Dose NR; used for 2.5 months	−13.6 kg	Hepatocellular; AST 261 U/L ALT 500 U/L TBil NR	5 (possible)	Discontinued; rechallenge then stopped permanently	2 months	Complete recovery
Fontana 2024 (64/M) [10]	Type 2 diabetes mellitus, hypertension, and hypercholesterolemia; ramipril, hydrochlorothiazide, and rosuvastatin; metformin, empagliflozin discontinued before tirzepatide initiation	2.5 mg weekly for 6 weeks	NR	Hepatocellular with cholestatic features; AST 629 U/L ALT 1093 U/L ALP 447 U/L TBil 7.1 mg/dL	NR	Discontinued	6 weeks	Complete recovery
Abdullah 2024 (24/F) [11]	Obesity; an oral contraceptive pill (Gynera)	Escalating doses over 5 months (2.5–12 mg)	−21 kg (27%)	Hepatocellular; AST 6712 U/L ALT 6212 U/L TBil 7.3 mg/dL	5 (possible)	Discontinued; ICU admission and supportive care	10 days	Improved
Terrington 2024 (33/F) [19]	Obesity; concomitant medications not reported	5 mg weekly for 4 months, followed by 10 mg weekly for 3 weeks (total duration, 4.5 months)	−25 kg	Hepatocellular; AST 4834 U/L ALT 2783 U/L TBil 4.6 mg/dL	NR	Discontinued; ICU admission and supportive care	24 days	Complete recovery
Ahmed 2025 (30 s/F) [13]	Obesity; no concomitant medications	2.5 mg weekly for 1 month, followed by 5 mg weekly for 2 months	−12 kg (14%)	Hepatocellular; AST 300–400 U/L ^§^ ALT 300–400 U/L ^§^ TBil NR	7 (probable)	Discontinued	4 weeks	Improved
Saleem 2025 (57/F) [20]	Type 2 diabetes mellitus and history of uterine cancer treated with total abdominal hysterectomy; concomitant medications not reported	Dose NR; used for 3 months	NR	Reported as cholestatic hepatitis; AST 337 U/L ALT 791 U/L ALP 141 U/L TBil 15.6 mg/dL	NR	Discontinued	NR	Improved
Phox 2025 (76/F) [16]	Obesity, hypothyroidism, major depressive disorder, hyperlipidemia, and prediabetes; aspirin, amlodipine, atorvastatin, fluoxetine, fluticasone nasal spray, levothyroxine, losartan, meloxicam, metformin, and pantoprazole	2.5 mg weekly for 4 weeks, followed by 5 mg weekly for 4 weeks	−7.3 kg	Mixed; AST 515 U/L ALT 486 U/L TBil 3.0 mg/dL	NR	Discontinued	2 months	Complete recovery
Mahmoud 2025 (19/F) [12]	BMI 28.1 kg/m^2^; concomitant medications not reported	5 mg weekly for 2 months	NR	Acute liver failure; detailed laboratory values NR	NR	Liver transplantation	NR	Liver transplantation
Spaeth 2026 (60/F) [18]	Obesity, type 2 diabetes mellitus, hypertension, hyperlipidemia, asthma, and MASLD; atorvastatin, amitriptyline, losartan/hydrochlorothiazide, mepolizumab, and famotidine; prior tolerance of semaglutide and liraglutide	2.5 mg weekly for 3 weeks	NR	Hepatocellular; AST 1466 U/L ALT 1562 U/L ALP 334 U/L TBil 1.6 mg/dL	7 (probable)	Discontinued	24 days	Complete recovery
Cao 2026 (20 s/F) [15]	Obesity, depression; venlafaxine, nortriptyline, and an oral contraceptive pill	2.5 mg weekly, 3 doses over 4 weeks	NR	Hepatocellular; AST 893 U/L ALT 1850 U/L TBil 0.7 mg/dL	NR	Discontinued	4 weeks	Complete recovery
Sudhir 2026 (42/F) [17]	Clinical background not reported; no concomitant medications	Dose NR; used for 3 months	NR	Hepatocellular; cholestatic features on histology; AST 697 U/L ALT 1155 U/L ALP 213 U/L TBil 3.7 mg/dL	6 (probable)	Discontinued	1 month	Improved
Alqarni 2026 (32/F) [14]	Obesity; no concomitant medications	Escalating doses over 20 weeks (5–15 mg)	−27 kg (31%)	Hepatocellular; AST 196 U/L ALT 1271 U/L TBil 3.7 mg/dL	NR	Discontinued; supportive care	6 weeks	Complete recovery
Present case (36/M)	Obesity and moderate hepatic steatosis on CT; no concomitant medications	2.5 mg weekly for 4 weeks, followed by one dose of 5 mg	−10 kg (11%)	Hepatocellular; AST 1336 U/L ALT 2626 U/L ALP 91 U/L TBil 4.0 mg/dL	7 (probable)	Discontinued; supportive care	2 weeks	Improved

NR, not reported; AST, aspartate aminotransferase; ALT, alanine aminotransferase; ALP, alkaline phosphatase; BMI, body mass index; TBil, total bilirubin; RUCAM, Roussel Uclaf Causality Assessment Method; MASLD, metabolic dysfunction-associated steatotic liver disease; ICU, intensive care unit. ^§^ Values approximated from graphical data. ALP values are presented only when reported in the original publication. Injury patterns and RUCAM scores were recorded as reported by the original authors and were not recalculated retrospectively when sufficient information was unavailable.

## Data Availability

The data presented in this case report are available from the corresponding author on reasonable request.

## Supplementary Materials

The following supporting information can be downloaded at: https://www.mdpi.com/article/10.3390/diagnostics16152359/s1, CARE Checklist for the present case report.

## Abbreviations

The following abbreviations are used in this manuscript:

GIP	Glucose-dependent insulinotropic polypeptide
GLP-1	Glucagon-like peptide-1
DILI	Drug-induced liver injury
RUCAM	Roussel Uclaf Causality Assessment Method
MASLD	Metabolic dysfunction-associated steatotic liver disease
BMI	Body mass index
ALT	Alanine aminotransferase
AST	Aspartate aminotransferase
INR	International normalized ratio
IgM	Immunoglobulin M
CT	Computed tomography
NR	Not reported
TBil	Total bilirubin
ALP	Alkaline phosphatase
ICU	Intensive care unit
IRB	Institutional Review Board
