# Supplementary material for: Tirzepatide-Associated Severe Hepatocellular Injury: A Case Report and Diagnostic Considerations
Source: Diagnostics (Basel). 2026 Jul 27;16(15):2359. doi: 10.3390/diagnostics16152359 (PMC13465100; doi:10.3390/diagnostics16152359)
Supplement: Supplementary file 1 [file diagnostics-16-02359-s001.zip › diagnostics-4354366-supplementary.pdf]

CARE Checklist of information to include when writing a case report

CARE case report guidelines

(cc) BY-NC-ND

Topic	Item	Checklist item description	Reported on Line
**Title**	1	The diagnosis or intervention of primary focus followed by the words "case report"	1-3
**Key Words**	2	2 to 5 key words that identify diagnoses or interventions in this case report, including "case report"	33-34
**Abstract (no references)**	3a	Introduction: What is unique about this case and what does it add to the scientific literature?	10-15, 24-29
	3b	Main symptoms and/or important clinical findings	15-21
	3c	The main diagnoses, therapeutic interventions, and outcomes	21-23, 29-32
	3d	Conclusion—What is the main "take-away" lesson(s) from this case?	29-32
**Introduction**	4	One or two paragraphs summarizing why this case is unique (**may include references**)	36-67
**Patient Information**	5a	De-identified patient specific information.	69, 72-81
	5b	Primary concerns and symptoms of the patient.	69-71
	5c	Medical, family, and psycho-social history including relevant genetic information	72-77
	5d	Relevant past interventions with outcomes	72, 78-81
**Clinical Findings**	6	Describe significant physical examination (PE) and important clinical findings.	82-86
**Timeline**	7	Historical and current information from this episode of care organized as a timeline	78-81, 87-132
**Diagnostic Assessment**	8a	Diagnostic testing (such as PE, laboratory testing, imaging, surveys).	82-108
	8b	Diagnostic challenges (such as access to testing, financial, or cultural)	105-108
	8c	Diagnosis (including other diagnoses considered)	112-113, 143-155
	8d	Prognosis (such as staging in oncology) where applicable	N/A
**Therapeutic Intervention**	9a	Types of therapeutic intervention (such as pharmacologic, surgical, preventive, self-care)	114-119
	9b	Administration of therapeutic intervention (such as dosage, strength, duration)	78-80, 114-119
	9c	Changes in therapeutic intervention (with rationale)	114-119
**Follow-up and Outcomes**	10a	Clinician and patient-assessed outcomes (if available)	115-123, 134-140
	10b	Important follow-up diagnostic and other test results	115-123
	10c	Intervention adherence and tolerability (How was this assessed?)	78-80, 134-140
	10d	Adverse and unanticipated events	69-71, 87-94
**Discussion**	11a	A scientific discussion of the strengths AND limitations associated with this case report	143-155, 221-233
	11b	Discussion of the relevant medical literature **with references**.	157-219
	11c	The scientific rationale for any conclusions (including assessment of possible causes)	143-155, 188-203
	11d	The primary "take-away" lessons of this case report (without references) in a one paragraph conclusion	221-233
**Patient Perspective**	12	The patient should share their perspective in one to two paragraphs on the treatment(s) they received	133-140
**Informed Consent**	13	Did the patient give informed consent? Please provide if requested	Yes ☒ No ☐
